# Modeling a primate technological niche

**DOI:** 10.1038/s41598-021-01849-4

**Published:** 2021-11-30

**Authors:** Jonathan S. Reeves, Tomos Proffitt, Lydia V. Luncz

**Affiliations:** Max Planck Institute for Evolutionary Anthropology, Technological Primate Research Group, Deutscher Platz 6., 04103 Leipzig, Germany

**Keywords:** Anthropology, Archaeology, Behavioural ecology

## Abstract

The ability to modify the environment through the transport of tools has been instrumental in shaping the evolutionary success of humans. Understanding the cause-and-effect relationships between hominin behavior and the environment ultimately requires understanding of how the archaeological record forms. Observations of living primates can shed light on these interactions by investigating how tool-use behaviors produce a material record within specific environmental contexts. However, this requires reconciling data derived from primate behavioral observations with the time-averaged nature of the Plio-Pleistocene archaeological record. Here, we use an agent-based model to investigate how repeated short-distance transport events, characteristic for primate tool use, can result in significant landscape-scale patterning of material culture over time. Our results illustrate the conditions under which accumulated short-distance transport bouts can displace stone tools over long distances. We show that this widespread redistribution of tools can also increase access to tool require resources over time. As such, these results elucidate the niche construction processes associated with this pattern of tool transport. Finally, the structure of the subsequent material record largely depends on the interaction between tool transport and environmental conditions over time. Though these results have implications for inferring hominin tool transports from hominin archaeological assemblages. Furthermore, they highlight the difficulties with connecting specific behavioral processes with the patterning in the archaeological record.

## Introduction

The human ability to habitually manipulate and reorganize landscapes is the most ubiquitous example of niche construction on earth^1^. Evidence of this trait can be seen as early as the Plio-Pleistocene when hominins began directly moving stones over of kilometers to gain access to otherwise inaccessible resources^2–8^. Over time, accumulations of discarded stone material at various locations would become sources of tools themselves, enhancing the availability of raw material across the landscape^9,10^. This interaction between tool behavior and the environment has been argued to enhance access to raw materials as well as influenced the mobility of hominins over the long term^4,9,11^. While these interactions may have been potent drivers in the evolution of hominin behavior^5^, our understanding of this process ultimately relies on understanding how the archaeological record forms^12–16^.

Traditionally our understanding of the behaviors associated with the formation of stone tool assemblages is derived from hunter-gatherers^17–21^. However, primate studies have generated new hypotheses regarding hominin behavior and the formation of Plio-Pleistocene sites^22–28^. Western chimpanzees (*Pan troglodytes verus*), for example, transport stone hammers to facilitate the cracking of nuts^22–24,29–31^. In contrast with early hominins^2,6,8,32–36^, primate stone tools are moved comparatively shorter distances^22,27,29,31^. Individual bouts of hammer transport are normally expedient and occur when stone and nut-bearing trees are conveniently situated near one another^29,31^. While each tool transport bout is comparatively short, researchers have argued that the accumulated effects of repeated transport over time could move tools over greater distances^1,2^. Studies show that stone hammers used for *Panda oleosa* nut cracking have been documented as far as two kilometers from the nearest known source of naturally occurring stone^31^. Landscape-scale patterns of hammer mass and utilization have been shown to follow distinct distance-decay patterns that are often found in the early Pleistocene record^8,31,35^. The accumulation of stone hammers at nut-cracking localities has been argued to increase the number of tool use opportunities at any given site, creating a tool-using niche^37,38^. These lines of evidence suggest that the primate model of stone tool transport is a potentially relevant analog for the formation of the Plio-Pleistocene record^22^.

The primate behavioral record is not equivalent to the early archaeological record. The majority of early hominin archaeological sites record the accumulation of behavioral events over hundreds, if not thousands, of years^6,35,39,40,40–43^ whereas modern primate studies represent significantly shorter time frames^29,44^. When considering longer time frames, tools eventually break, become unusable due to repeated reuse and therefore will no longer be moved^45^. In addition, the number and location of resources that require tool use will inevitably change over time, influencing the patterning of the archaeological record^46^. With respect to nut-cracking, the locations of nut-bearing trees will change due to the death of old trees and the growth of new ones. The interaction of tool re-use and diachronic spatial patterning of trees may create disconnects between observed nut-cracking behavior and the chimpanzee archaeological record^45^. Archaeological investigations at the chimpanzee nut-cracking site of Panda 100 recovered no functional tools, even though chimpanzees were observed using stone hammers at the site up until the Panda tree died^45^. Thus, to understand the relevance of the primate tool transport model for hominin behavior, an understanding of the environmental conditions that facilitate long-distance movement of stone via aggregated small scale transport bouts, and how it structures the time-averaged archaeological record is needed. Such work can also identify the general mechanisms through which tool use behavior, the environment, and the formation of the archaeological record interact. Thus, this would also generate more nuanced expectations to investigate behavioral patterns and processes in the Plio-Pleistocene archaeological record.

However, such an endeavor requires an understanding of how behavioral and environmental processes produce patterns that emerge over timescales that cannot be observed in the natural world. Generative modeling provides a means by which to investigate how the interaction between the environment with known behaviors produces patterning in the material record over time^47–50^. Here, we present a spatially explicit agent-based model (ABM), to address the primary research questions: (1) Under what environmental conditions do repeated bouts of small-scale transport result in long-distance movement of tools, and (2) how does such transport structure the resulting material record over time? In doing so, we modeled short-distance tool transport after real-world observations of chimpanzee nut-cracking behavior^29,51^ in landscapes with varying numbers of resources requiring tool use and raw material sources to understand their effect on the displacement of tools. In doing so, we illustrate the conditions in which cumulative short-distance bouts transport move tools distances far greater than during any observed individual transport event. Such insights are important as there is a growing consensus that extant non-human primate tool-use may have been the precursor to the current earliest physical evidence of tool-use and transport in the archaeological record of hominins^46^.

## Materials and methods

The model was designed and implemented using Python 3 and the ABM library Mesa^52^. The version of the model present here is actively maintained and available for download at a git-hub page (see SOM). A full description of the variables, design, justification, and implementation of the model following Grimm et al.^53^ is provided as supplementary material. The model consists of a 250 × 250 grid-cell space that is populated with four entities: *Primates*, *Sources*, *Trees*, and *Pounding Tools*. This grid space can be thought of as a forest that *Primates* move through, transporting stone tools over small distances as they encounter locations where they can crack nuts. *Primates* are agents who move around the landscape using tools to crack nuts at any opportunity. *Sources* reflect places where *Pounding Tools* can be acquired (e.g. inselbergs and cobble beds). *Sources* also possess a fragility score which determines how likely an acquired *Pounding Tool* is to break during use (see below).

*Pounding Tools* represent hammers used to crack nuts and have the attributes size and fragility. The size of the *Pounding Tool* is determined by randomly drawing from a normal distribution with a mean and standard distribution equivalent to the mass (grams) of the Panda nut hammers recovered in the Tai Forest (5). The fragility of the *Pounding Tool* is inherited from the *Source* it was acquired from and, thus, determines the likelihood it will break during use and subsequently lose mass. Each tool has a baseline 25% chance of breaking plus its fragility score. For example, if the tool’s fragility score is 25, then its probability of breaking is 50%. When the *Pounding Tool* breaks the size of the tool decreases to simulate fragmentation associated with breakage. The amount that is subtracted from the original size is based on the observed size distribution of fragments detached from *Pounding Tools* during modern chimpanzee nut-cracking events in which most breakages result in the production of small fragments but in rare cases, fragments can also be large^45^. *Pounding Tools* can be continuously re-used (and reduced in size through use) until they are considered unusable when their size decreases below 2000 g. This size threshold is modeled after the smallest Panda nut cracking hammer in the Taï Forest^31^.

*Trees* represent locations of resources that can only be accessed through the use of tools. *Trees* exist only at fixed locations. Over time frames relevant to the formation of archaeological sites, however, the death and growth of trees can restructure where the resources are located^46^. To investigate the effect of this process on cumulative tool transport distances, the death of old *Trees* and the growth of trees at new locations are considered in the simulation. *Trees* increase in age by a unit of 1 after each time-step and will die when their age is equal to 10,000 time-steps. When a *Tree’s* age reaches 10,000 time-steps, tool use no longer occurs at this location. A new location within a 10 grid-cell radius is randomly chosen as a place for a new *Tree* to “grow.” This ensures that the number of trees remains constant throughout the simulation.

When the model is instantiated, *Trees*, *Sources*, and *Primates* are randomly placed within the grid-cell space. Each *Source* is randomly assigned a value of 0, 25, 50, or 75. To prevent every *Tree* from dying at the same time-step, *Trees* present at the start of the simulation are randomly assigned an age between 1 and 10,000. New *Trees* that grow after the model is initialized begin with an age of 0. The population of *Primates* was held constant at 100 for each run of the model. To investigate the effect of the density of resources requiring tool-use (*Trees*) on the distance *Pounding Tools* were moved from their *Source*, the number of *Trees* varied between 100, 500, 1000, or 2000. In addition, to examine the effect of raw material abundance on this pattern, the number of *Sources* was also varied between 10, 100, 500. To further understand the influence of changing *Tree* locations (due to death and growth) on *Pounding Tool* transport distance, we also varied whether *Trees* could change location. The duration of the model run is 75,000 time-steps. 75,000 time-steps allows for the majority of model runs to “fixation” in which *Pounding Tools* cannot be displaced any further from their *Sources*. In some cases, the model did not achieve fixation by 75,000 timesteps (see Fig. 2). However, 75,000 is used as a cut-off point as the additional information gained by allowing the model to reach fixation does not influence the general patterns described in the results.

A single time step is approximately equivalent to the amount of time it takes for an individual primate to carry out a nut-cracking episode. During each time-step, *Primates* move a length of 1 grid cell in a random direction. If the *Primate* moves into a grid-cell that neighbors or is occupied by a *Tree*, the *Primate* will check to see if a *Source* or *Pounding Tool* is within a radius of 2 grid-cells around its location. If there is none, then the *Primate* does nothing for the rest of the time-step. If a *Source* is within the search radius of the *Primate*, the *Primate* will acquire a *Pounding Tool* from this location. If a previously used *Pounding Tool* is found within the search area, then the *Primate* will re-use the *Pounding Tool* provided that it is 2000 g or greater in size. If multiple *Sources* and *Pounding Tools* are found within the search radius then the *Primate* will choose the *Pounding Tool* or *Source* that is nearest to its location. If multiple *Pounding Tools* or *Sources* are equally near, then the choice is random. The *Primate* then moves the acquired *Pounding Tool* to the location of the *Tree* or one of its eight neighboring grid-cells where it is used and discarded. This ensures that the maximum distance a *Source* and/or *Pounding Tool* can be from a *Tree* and still be moved is 3 grid-cells. Each time a *Pounding Tool* breaks during use, an additional *Pounding Tool*, representing the fragment (hereafter referred to as fragments), is discarded at its location.

During the simulation, data regarding the grid space, and the individual agents is recorded. The model monitors the number of tool-use locations that exist on the landscape during each time-step. A tool use location is defined as a *Tree* that is within a distance of 3 grid cells from a *Source* or a usable *Pounding Tool*. In iterations where *Trees* can die and grow, the locations *Trees* are recorded through time. In addition, each *Pounding Tool* records the *Source* that it originated, the number of times it was used, its initial size, its current size, as well as its location at the end of the simulation. At the end of the simulation, the model provides data on the location of each *Pounding Tool*, *Sources*, and *Trees* as well as their attributes. This provides a means to examine the relationship between where tool use occurs and the location of *Sources* and *Trees* from both systemic and archaeological perspectives.

## Results

### Environmental conditions of tool displacement

**Figure 1:**
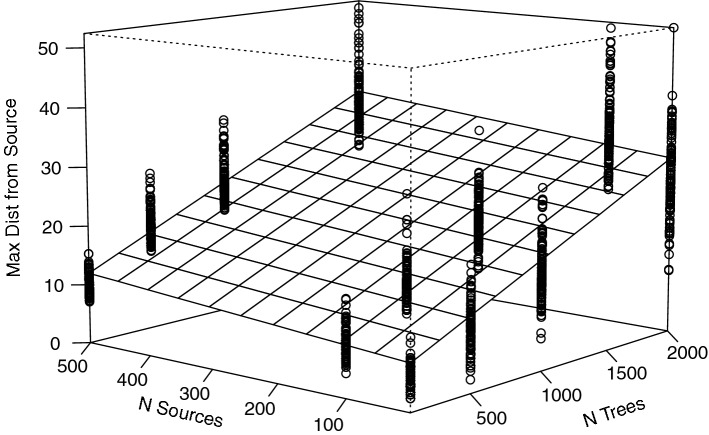
A two-way plot illustrating the effect of the number of *Trees* and number of *Sources* on the maximum distance *Pounding Tools* move from its source. An ordinary least squares regrsssion shows that the number of *Sources* and *Trees* have a positive effect on the distances *Pounding Tools* can be moved (R^2^: 0.76). The number of *Sources* has a marginal but significant effect on the maximum distances *Pounding Tools* move but this effect increases with the number of *Trees* (p-value < 0.0001). Increasing the number of *Trees* has a much greater effect on the maximum distance a *Pounding Tool* can move from its *Source* (p-value < 0.0001). The points represent the specific values associated with each iteration for a given parameter combination.

**Figure 2:**
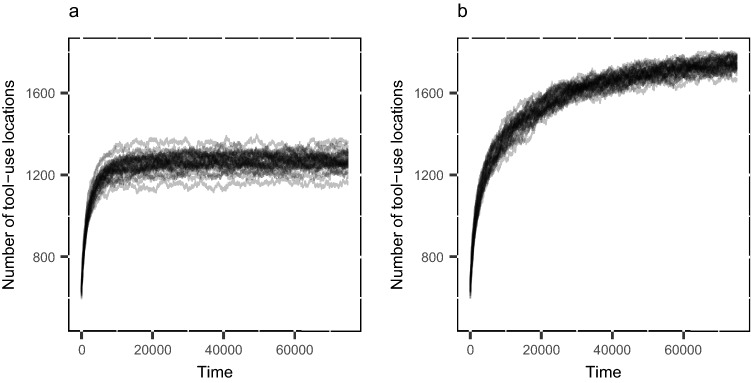
The number of tool-use locations per time-step through time when the number of Sources is 500 (high-density) and the number of Trees is 2000 (high-density). The number of tool-use locations increases (y-axis) due to the repeated displacement of *Pounding Tools* away from their *Sources*. (**A**) *Tree* locations do not change through time. (**B**) *Tree* locations change through time (see text). Each line represents an individual iteration of the model. Under these conditions, the number of tool-use locations plateaus at a higher value when Trees change locations over time. It is expected that the number of *Trees* that become accessible for tool use would eventually reach the maximum number of Trees in the model. However, this remains unclear given that the simulation ended at 75,000 time-steps.

At the beginning of each model run, tool-use can only occur in places where a *Tree* is located within 3 grid cells of a *Source*. Simply increasing both the number of *Sources* and/or *Trees* increases the number of places where tool use initially is possible (SOM Fig. S1: left, Kruskal–Wallis, chi-squared: 225.4, *p*-value < 2.2e−16). After 75,000 time-steps, however, we find that *Pounding Tools* were moved to *Trees* greater than 3 grid cells from the nearest source in 95% of the runs. When a *Pounding Tool* is moved from a *Source*, it becomes a secondary source of material for tool use at other *Trees*, allowing it to be move from *Tree* to *Tree* away from its *Source*. In conditions where the number of *Trees* is low, *Pounding Tools* are not displaced far from their *Sources*. *Pounding Tools* move greater maximum distances when *Trees* are more plentiful (Fig. 1, Kruskal–Wallis, chi-squared: 1667, *p*-value < 2.2e−16). In conditions with high *Tree* densities, the distance a *Pounding Tool* is moved becomes a function of time. The longer the model runs, the farther *Pounding Tools* will be displaced from their *Source* (Fig. 2). This redistribution of tools consequently increases the number of tool-use locations across a wider landscape. At the end of 88% of all runs, there are more places where tool-use can occur than at the beginning (Fig. 2, see SOM Table 3 for cases where is this is not the case).

The landscape-scale redistribution of *Pounding Tools* is facilitated by the use-lives of *Pounding Tools*. The small size of detached fragments in combination with their potentially large size allows *Pounding Tools* to be moved and used 171 to 835 times before exhaustion. These long use-lives allow *Pounding Tools* regardless of fragility to be moved substantial distances from their *Source*. Nevertheless, material that break less tend to move greater maximum distances under conditions when (SOM Fig. S2). The interplay between changing *Tree* locations over time and the extended use-life of *Pounding Tools* further facilitates the distribution of tool materials across the landscape. If *Tree* locations are static, the increase in the number of tool-use locations, over time, eventually plateaus far below the number of *Trees* in the grid space (Fig. 2). By contrast, when *Tree* locations change through time, a greater number of tool-use locations become available. The number of tool use locations would likely continue to increase until all *Trees* in the simulation became available for tool use (Fig. 2, SOM Figs. S3, S4).

### Material signature

**Figure 3:**
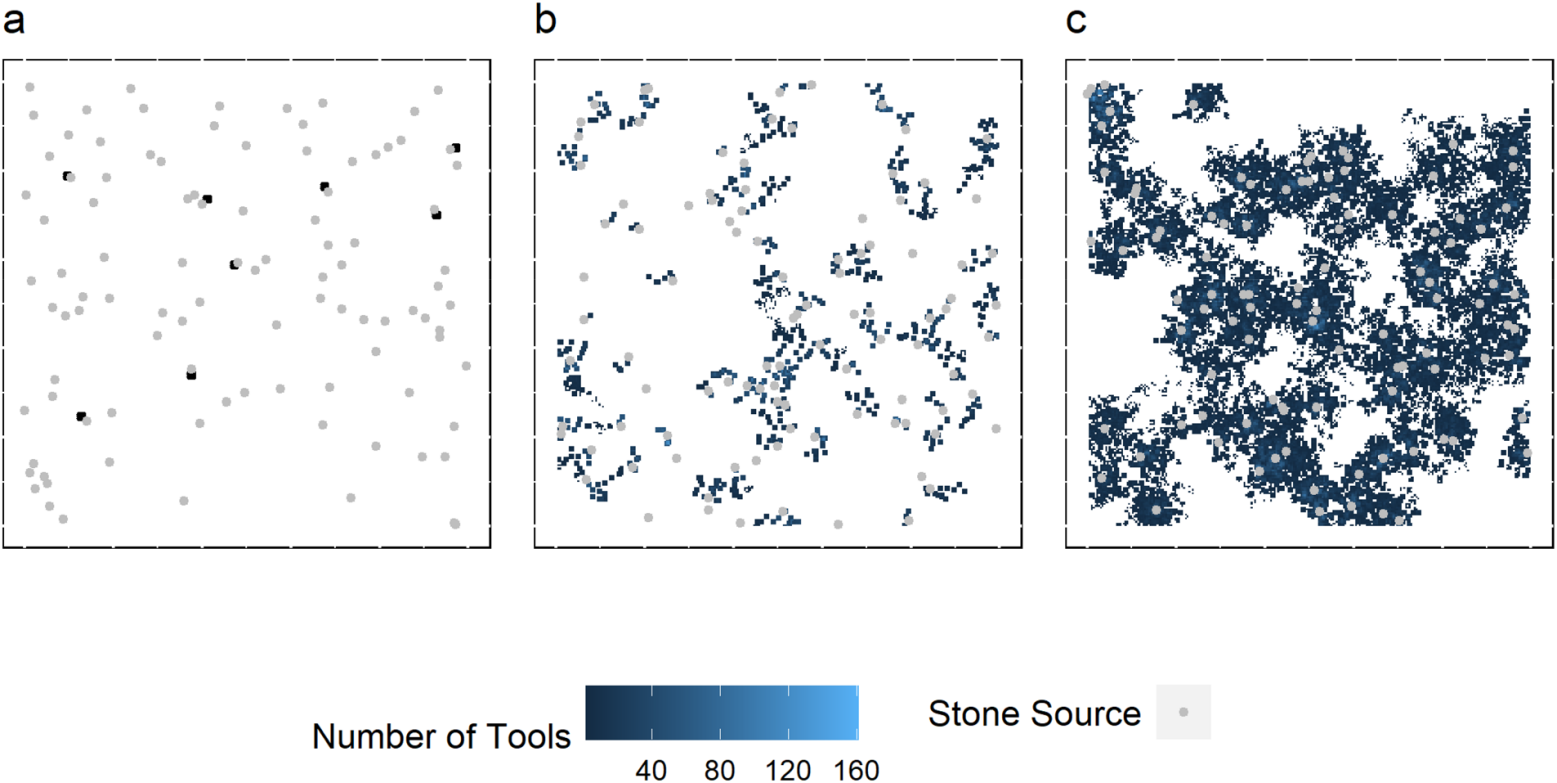
(**a**) The archaeological record when there are 100 *Sources* and 100 *Trees*. Notice that the subsequent archaeological record forms extremely localized patches of material. (**b**) The material record when there are 100 *Sources* and 2000 *Trees*. This material record is becoming more widespread but remains localized. (**c**) The archaeological record when there are 100 *Sources* and 2000 *Trees* where *Tree* locations change over time. Notice how the material record becomes substantially more widespread under these conditions.

The modeled transport behavior creates a material record that is comprised predominantly of fragments detached from *Pounding Tools* but also exhausted and usable *Pounding Tools* in substantially smaller quantities. The extent to which *Pounding Tools* can be displaced from their *Sources* influences the structure and composition of material assemblages at the landscape scale. When the number of *Trees* is low, material assemblages create localized concentrations at *Trees* nearest to *Sources* as they cannot be displaced further (Fig. 3a, SOM Figs. S6, S7). As a result, usable *Pounding Tools,* exhausted *Pounding Tools,* and fragments are all found within these localized concentrations.

**Figure 4:**
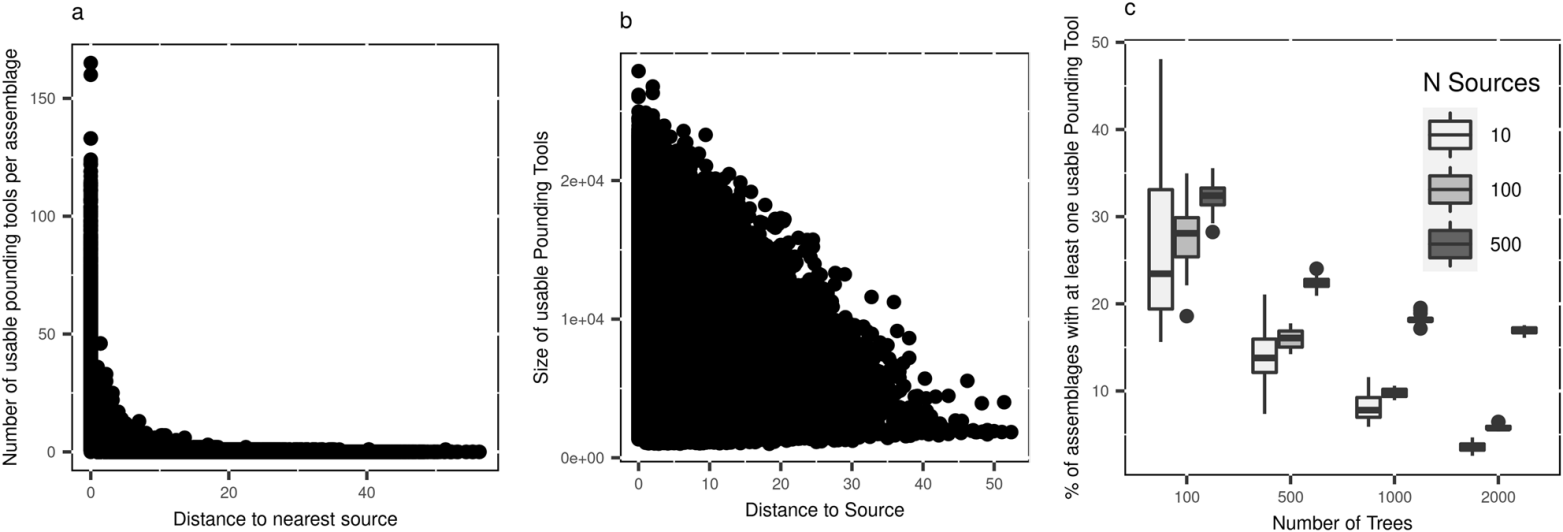
(**A**) Scatter plot showing the relationship between the number of usable *Pounding Tools* contained within a single grid cell with the distance to the nearest *Source*. Since grid cells are not associated with a *Source* their relationship to *Sources* is expressed as the minimum distance. (**B**) The relationship between usable *Pounding Tool* size (grams) and distance to its *Source*. (**C**) The effect of the environment on the proportion of grid-cells where *Pounding Tools* are represented within an individual assemblage. Increasing the number of *Sources* increases the percentage of assemblages that contain *Pounding Tools*. Increasing the number of *Trees* or allowing the *Trees* to change locations substantially reduces the proportion of assemblages that contain useable tools (See SOM Fig. S8). Plots (**A**) and (**B**) show run where the number of *Sources* is 100, the number of *Trees* is 2000 and *Trees* can change location. See SOM Figs. S5, S6, and S7 for other parameter combinations.

As the number of *Trees* increases, so do the distances that *Pounding Tools* can move, causing the material record to become more widespread (Fig. 3b). The changing locations of *Trees* have the greatest effect on the distribution of the archaeological record across space (Fig. 3c). In addition, this wider displacement of *Pounding Tools* also causes the density of discard material and the size of *Pounding Tools* to decrease, following distance-decay pattern, as the distance from a source increases (Fig. 4, SOM Figs. S5, S6). The total number of materials (*Pounding Tools* and fragments) per grid cell is greatest at locations nearest to *Sources* and decreases exponentially as distance between an assemblage and the nearest source increases (Fig. 4a, SOM Fig. S5). In addition, *Pounding Tool* size is also negatively correlated with the distance to *Source* locations (Fig. 4b, SOM Fig. S6). The representation of useable *Pounding Tools* is also influenced by the extent to which tools can be displaced. Under conditions in which tools move greater distances, as little as 2.5% of the total assemblages. The preponderance of assemblages lacking *Pounding Tools* is explained in part by the fact that while usable *Pounding Tools* may be transported to a different tree, small fragments are not and remain at the location where they were produced.

## Discussion

Primate tool use and material culture have become an increasingly useful framework for investigating the behavior of past hominins^25,28,31,38,45^. However, maximizing the utility of primate models requires reconciling the relatively brief temporal scale of ethological studies with the time-averaged nature of the early hominin behavioral record. By modeling the accumulated effects of primate tool transport events, it is possible to identify conditions that promote the landscape wide displacement of tools over time. In doing so, we can illustrate the potential effects of primate tool transport on the landscape distribution of tool-use locations as well as the archaeological patterns that emerge due to time-averaging.

The results of our model highlight the capacity for short-distance tool transport events, repeated over many generations, to modify the availability of stone at a landscape scale. *Primates* only engaged in tool-use when usable *Pounding Tools* coincided in time and space (i.e. within 3 grid cells) with *Tree* locations. We found that when *Tree* density is low, *Pounding Tools* seldom moved beyond the few grid cells from their sources. However, the long-use of pounding tools permits repeated short-distance transports to move farther away from their sources when *Trees* and *Sources* exist at higher densities. In turn, this increases the number of locations where tool use can occur during the simulation, decoupling the availability of stone from the natural environment over time. Furthermore, this process can work in tandem with the changing distribution of tool-use localities over time. This further increase the spread of tool material and access otherwise inaccessible resources across the landscape. The number of places where tool use can occur on the landscape at time-step 0 is markedly different from those present at time-step 75,000.

The model is not a direct reproduction of any single primate context. However, the similarities between the model and the environmental context of primate nut-cracking allow us to discuss its results in the context of primate behavior. These results highlight the niche constructing capacity of primate tool-using behaviors as it demonstrates how the residue of previous tool-using events can act as facilitators of future events in places where tool-use was previously not possible^38^. While it has been suggested that the aggregation of hammers at specific sites creates new tool use opportunities for future generations^38^, the relatively short tool transport distances limits tool use to places where both tool and food resource coincide in space^23,54^. Our model, however, provides a proof-of-concept that primates may possess the capacity to increase the number of tool use opportunities through the landscape-scale redistribution of tool material over time.

Increasing tool-use locations would also increase access to potentially valuable food resources which could help mitigate periods where other food resources are scarce. Furthermore, this increase in tool-use locations also enhances the potential for the acquisition of tool-using skills^38,54,55^. As a result, this process may ensure the continuation and spread of tool-using behaviors over time. Given the long use-life of percussive stone tools, it may be that the redistribution of pounding tools generates feedback in which future generations of primates inherit a landscape where opportunities for tool use are greater than in the past^38^. Since tool use is considered to be socially learned^56^, our results may also imply that primates can increase their accessibility to resources through a culturally learned behavior though this would require further testing empirical data.

The processes described by the model also help to better understand the potential mechanisms responsible for the disconnects between observed primate behavior and the material records they produce. For example, the chimpanzees of the Taï forest have been observed moving Panda nut hammers between nut trees over multiple decades^29,51^. However, it has been impossible to reconcile the widespread distribution of Panda nut hammers in the Taï forest with the relatively short tool transports observed in this field^31^. Given the similarities between the distribution of resources in the model and the Taï forest, our results provide a proof of concept that given enough time, the cumulative effects of small-scale tool transport can produce a landscape-scale pattern (as suggested by Luncz et al.^31^). This notion is further supported by the fact that the spatial distribution of size and damage intensity of these hammerstones is consistent with the distance-decay relationship described in the model presented here. The described processes also provide a potential explanation for the absence of hammerstones from the chimpanzee archaeological assemblage at Panda 100^45^. Since functional hammerstones can act as raw material sources for nearby tool-using locations, hammerstones can be transported to nearby trees instead of remaining at a single locus. As a result, hammers are less likely to enter the record at every location where nut-cracking occurred. In this light, the processes that play an active role in structuring the material record can sometimes obfuscate intuitive connections between past behaviors and their material correlate.

These results have implications for the understanding of hominin behavior and the role that niche construction processes may have played in the formation of the Plio-Pleistocene archaeological record^1,5,10,57^. Processes described in our model imply that Plio-Pleistocene hominins could increase their access to resources as soon as they began repeatedly transporting durable materials even over short distances. Researchers have often argued that the capacity to transport material over large distances (up to 11 km) at a time is fundamental to Oldowan hominin behavior^2,3,8^. This work shows that specific conditions exist in which long distance transport events are not needed to move tools across landscapes. In light of this work, it may be possible, on a preliminary basis, to suggest that the landscape scale patterning in the Oldowan is the aggregation of hundreds if not, thousands of years of short-distance transport events.

It is important to consider, however, that repeated short-distance transport events are most likely to result in the displacement of tools over longer distances where resources are abundant. While the narrow set of conditions illustrated by the model maybe broadly applicable to stone tool-using primate populations, its direct applicability to hominins may be less so. It remains unclear whether the landscape distribution of resources exploited by tool-using hominins would have facilitated the movement of tools in the Oldowan, in the same way, as it likely does in the Taï Forest. Moreover, results indicate that this pattern of tool transport is only possible with tools that have extremely long use-lives. Oldowan core and flake technology are utilized in a fundamentally different way to primate percussive tools and may have shorter use-lives. Therefore, understanding how interactions between the environment, tool use-life, and tool transport increases access to resources will provide new insights into the adaptive benefits of tool-use within the hominin clade.

Finally, the results of our model illustrate the difficulty in inferring dynamic processes from time-averaged archaeological patterns. The material records of the modeled processes vary in their structure and composition depending on the availability of resources on the landscape. In scenarios where the potential tool-using locations are few, lithic assemblages remain localized, with unexhausted Pounding Tools present within each assemblage. However, when Pounding Tools were moved over large distances, the landscape signature becomes widespread. This results in the formation of distance-decay relationships between the location of sources and the density of artifacts, as well as the number and size of Pounding Tools. The proportion of assemblages that contain at least one usable Pounding tool after 75,000 time-steps decreases as the density of Trees increases. This is due, in part, to an increase in the number of assemblages. Thus, not only is this behavior represented by a wide range of varying archaeological patterns but the repeated movement of usable tools, actively leads to the under -representation of the most archaeologically recognizable components of the behavior.

Inferences regarding human–environment interactions are often predicated on the spatial structure of and variation within the archaeological record^35,36,57–60^. Here we show that vastly different spatial patterns can be created by varying the density and stability of resources alone without changing the underlying behavior. In cases, where resources requiring are dense, archaeological measures such as distance to the raw material source, and relationships between tool-utilization and transport distance become a function of time. Therefore, a single behavior can produce vastly different patterns depending on the amount of time-averaging and the density of resources in the environment. This work illustrates how time-averaged archaeological patterns emerge due to the interplay between the environment, behavior, and time^19,46^. While linking the patterning of the material record to specific behaviors that produced it remains difficult, the lithic assemblages of the modeled percussive behavior are much simpler than what is found in the hominin archaeological record. Thus, further investigations that are more tailored specifically to the variability observed in the hominin archaeological record are needed. Nevertheless, developing methods that treat archaeological patterning as an emergent phenomenon is critical for better linking the interplay of behavioral and environmental process with the patterns described in the archaeological record.

## Conclusion

Drawing behavioral inferences from the archaeological record require robust referential frameworks^61^. Although primates provide a useful analog for investigating hominin behavior, it is difficult to translate observable behaviors into a time-averaged archaeological patterns. Agent-based modeling provides a means to investigate how the interaction between behavioral and environmental processes may influence the formation of material records. The results of our study the conditions in which repeated short-distance tool transport bouts generates causal feedback with resource density that can promote the spread of tool material and increase the number of opportunities for tool-assisted foraging at the landscape scale. The strength of our approach lies in not only highlighting the potential niche constructing capacity of primate tool transport but also in explicitly describing the processes likely responsible for patterning of the material record. Although the links between such processes and archaeological patterns are complex and seemingly opaque, such work is critical to better establishing connections between niche constructing processes in the past and the record from which we interpret behavior.

## Supplementary Information

Supplementary Information 1.

Supplementary Information 2.
