# Supplementary material for: Modeling a primate technological niche
Source: Sci Rep. 2021 Nov 30;11:23139. doi: 10.1038/s41598-021-01849-4 (PMC8632893; doi:10.1038/s41598-021-01849-4)
Supplement: Supplementary file 1 — Supplementary Information 1. [file 41598_2021_1849_MOESM1_ESM.docx]

**Supplementary Material 1: Overview, Design Concepts and Details for the Agent-Based Model presented in “Modeling a Primate Technological Niche”**

This model description follows the ODD protocol for agent-based models ^1^ and is provided as supplementary material to the paper entitled “Modeling a Primate Technological Niche”. The model was written in Python 3.7 using the MESA agent-based modeling library ^2^. The source code for this model is actively maintained on the Author’s git-hub page and can be downloaded by following this [link](https://github.com/reevesj191/Panda_Tool_Use_ABM).

**Purpose**

Primate tool-use is often considered to be expedient, involving transport of tools over short distances. Nevertheless, stone tools, used by West African chimpanzees, have been documented kilometers from where they naturally occur ^3^. However, single movements of stone over long distances have never been observed ^3–5^. Therefore, the purpose of this model is to investigate the mechanisms through which repeated of short distance transport events move tools across large distances. We attempt to provide insight into the cumulative effect of short-distance tool transport on the broader landscape by addressing the following questions in our experimental design: *Can the aggregate effects of repeated short distance transport of stone lead to the large-scale movement of stone across space? If so, under what environmental circumstances does this occur? What landscape scale signature does this behavioral process leave behind? How does this process influence the number of opportunities for tool-use on a given landscape?*

**Entities, State Variables, and Scale**

The model is spatially explicit. The environment is comprised of a 2-dimensional 250 x 250 grid cell space. The 2-D space is not wrapped into a torus and thus has discrete boundaries around its edges. The space is inhabited by four types of agents: Primates, Sources, Pounding Tools, and Trees. Time is represented with discrete steps called “Time-Steps”. Wach time-step encompasses enough time for a Primate to move a distance of 1 grid cell or carry out a “tool transport” episode in which they will move more than 1 grid cell in a time step.

**Primate** agents are akin to West African chimpanzees. Primates are able to execute five methods: *Move, CheckForTree, CheckForStone, SelectStone, UseStone, BreakStone* (see descriptions of each in the Submodels section below). Primates also possess eight attributes: *pos*, *search_radius, NutTreeLoc, StoneLoc, Tool_size, Tool_id, Source_id, rm_qual.*

*pos:* (x,y) coordinates reflecting the current location of the agent. This attribute is recorded as the variables x and y in the data tables outputted by the model.

*search_radius:* the distance that the primate will look beyond its current location to determine if a Source or Pounding Tool can be acquired. This value is set equal to the global variable *search_rad*. This parameter is set at 2 for all model runs.

*NutTreeLoc:* (x,y) coordinates corresponding to the location of the a **Tree** agent encountered by the Primate.

*StoneLoc:* (x, y) coordinates representing the location of a pounding tool that has been selected for tool transport (See CheckForStone method in sub models)

*Tool_size:* The mass (grams) of the **Pounding Tool** in possession of the agent.

*Tool_id*: A six-character alpha-numeric value that is unique to the tool that the PrimAgent currently possesses.

*Source_id*: The unique identifier of the originating source that the **Pounding Tool** in the agent’s possession.

*rm_qual:* The quality of the selected **Pounding Tool**. This variable is inherited from the rm_quality attribute of the **Pounding Tool**. This attribute has a value of -1 when the Primate has not acquired a Pounding Tool.

***Pounding Tools*** are analogous to the hammers or anvils that primates use to crack nuts. They have the following 11 attributes: *parent_id, tool_id, pos, Tool_size, original_size, active, source_id, rm_quality ts_born, ts_died, n_uses*

*parent_id*: New pounding tools can form when a **Pounding Tool** is broken during use (See PrimAgent method ***BreakStone***. If this occurs, the newly generated **Pounding Tool** inherits the *tool_id* of the stone broken as the *parent_id.*

*tool_id*: An 8-digit alphanumeric string that acts as a unique identifier for the tool.

*pos:* (x,y) coordinates reflecting the current location of the **Pounding Tool**. This attribute is recorded as x and y in the data tables outputted by the model.

*tool_size:* A numeric value referring to the size of the tool. This is assigned when a **Pounding Tool** is acquired from a source location. The size of the tool determined by randomly drawing a value from a normal distribution with the mean and standard deviation equivalent to the size distribution of known Panda nut tools.

*original_size*: A numeric value (grams) referring to the starting size of the **Pounding Tool**.

*active*: A Boolean value that determines whether the tool can be re-used as a **Pounding Tool**. True means that it can still be used and transported. False means that the tool can no longer be used and transported.

*Source_id*: To the id of the source where the **Pounding Tools** originated. It is inherited from the source attribute ID.

*rm_quality*: a value inherited from the source agent it originated from determining the likelihood that pounding tool will break.

ts_born: an integer value corresponding to the specific time-step when **Pounding Tool** was generated.

ts_died: an integer value corresponding to the specific time step when the **Pounding Tool** became no longer available for transport.

n_uses: an integer value corresponding to the number of times a tool has been used.

***StoneSources*** are locations where Pounding Tools can be acquired (See ***PoundingTool*** *agent*). This agent possesses three attributes: *ID, rm_quality, pos.*

*ID:* A unique identifier inherited from the global model environment. It can be used to pair **Pounding Tool** agents to the source that they originated from.

*rm_quality*: an integer value of 0, 25, 50, 75. This determines how likely a stone is to break during use (See BreakStone submodel).

*pos:* (x,y) coordinates reflecting the current location of the agent. This attribute is recorded as x and y in the data tables outputted by the model.

***Trees*** are analogous to the nut bearing trees whose nuts require tool-use to access. These agents do not change location throughout the simulation. The model can be set so that trees age, die and new trees grow during the simulation. This agent class is able to execute two methods: *AmIAvailable* and AgeDieGrow*.* Trees also 7 attributes: *ID*, pos, *ts_born*, *ts*_*died*, *age*, *alive*, *available.*

*ID*: an integer value that acts as a unique identifier for each **Tree**

*pos:* (x,y) coordinates reflecting the current location of the agent. This attribute is recorded as x and y in the data tables outputted by the model.

*ts_born*: the time-step that the **Tree** is placed in the model space.

*ts_died*: the time-step that the **Tree** agent was removed from the model space,

*age*: an integer value that reflects how many time-steps the *Tree* has existed in the model space. In simulations where trees do not age and die, this value remains -1 where as when trees die and grow, this value will range from 0 to 10000.

*Alive*: True/False attribute refers to whether the Tree is active on the landscape.

**Global parameters** include: *run_id*, datetime, *num_agents, num_sources, num_nuttree, treesdie, max_ts, search_radius, exp_name, runs_path, sql, schedule, grid, timestep, running*

*run_id:* A randomly generated six character long alpha numeric string that is unique to the current iteration of the model.

*Datetime:* The time and date that the model run began.

*num_agents:* A user defined numeric that is passed to the model to set the number of **Primates** to be included in the model run.

*num_sources:* A user defined numerical value passed to the model to set the number **Sources** to be included in the model run.

*Num_nuttree*: A user defined numerical value passed to the model to set the number of **Trees** to be included in the model.

*Treesdie*: A user defined Boolean expression that determines whether **Trees** can die, and new ones can grow during the model run. True means that death and growth is possible. False means that **Trees** placed during the instantiation of the model remain unchanged for the duration of the run.

*max_ts*: A numeric value corresponding to the the number of time-steps that the number is required to run for.

*search_radius*: An integer value that reflects the distance that the user would like primate agents to search for stone. All iterations of the published model have this value set at 2.

*exp_name*: A user defined string that is the name of SQL database where the output of the model is saved.

*runs_path*: An automatically generated file pathway where the results of each model run is saved.

*sql:* An automatically generated file pathway referring to the SQL database where the runs will be saved.

*schedule:* All models written using MESA must have a schedule attribute. This refers to how the model will activate each agent during the simulation. Agent behaviors are scheduled using random activation.

*grid:* All models written using MESA must also have an attribute defining the type of grid space. The grid space in this model is set to multi grid.

*height:* A user defined numerical value determining the size of the model environment along the y-axis.

*width:* A user defined numerical value determining the size of the model environment along the x-axis.

*timestep:* An integer that indicates the current time-step of the model.

*tree_growth_deaths:* an integer value that refers to number of times a tree has died during the simulation ( see *AgeDieGrow* sub-model).

*running:* a True/False statement that indicates whether the simulation is running or note.

**Processing Overview and Scheduling**

During each time-step the Primate agent will carry out a series of behaviors that are conditional on specific circumstances. The Primate will move by randomly choosing one of its neighboring grid cells to move into (Submodel move). Then the Primate will execute the following behaviors. (1) The Primate agent will check to see a Tree exists within one of its neighboring grid-cells or the cell that it currently inhabits (Submodel CheckForTree). (2) If this is true, then the Primate will check to see if the if a Pounding Tool agent or a Source agent are within its search radius (Submodel CheckForStone). (3) If a Pounding Tool or Source exists within the search radius, the agent will move this stone to one of the neighboring grid-cells of the Tree or the location of the tree itself to use the tool (Submodels SelectStone and UseStone). (4) Pounding Tool attributes are then modified as a result of its use (Submodel BreakStone). If a Pounding Tool’s size drops below 2000 as a result of repeated uses, then Pounding Tool attribute active is set to False. If the global parameter *treesdie* has been set to True then Trees will increase their age by a unit of 1. If a Tree reaches an age of 10000, then it is removed from the grid space and a new tree with an age of 0 will be added at a different location. The model ends when the maximum number of time steps has been reached. At this point the global parameter *running* is set to false and the attributes of the model are exported as an SQL database. Subsequent analysis of the model output is carried out in R.

**Design Concepts**

Emergence

The landscape scale point patterns of Pounding Tools emerge from their repeated movement of Pounding Tools over time. Despite the fact that Pounding Tools never move more than 5 grid-cells at a time (see stochasticity), it is expected that Pounding Tools will move from the sources where they originate distances greater than 5-grid cells. It is also expected that this type of movement will also produce a special type of distance-decay point pattern where the number of pounding tools found in any given grid-cell will decrease as the as the distance to the nearest source increases. Pounding Tool movement also affects how various Pounding Tool attributes are partitioned across space. Under this pattern of movement, the size of the Pounding Tool attributes *tool_size* will decrease, and *n_uses* will increase as the distance to its origin source increases.

Stochasticity

Stochasticity can be found in many places throughout the initialization and implementation of the model. During initialization, the locations of Sources, Trees and the starting locations of Primate agents are chosen at random. Each source is randomly assigned an *rm_quality* value of 0, 25, 50, or 75. If the global parameter *treesdie* is set to True then the *age* attribute for each Tree is assigned a random value between 1 and 10000. During each timestep, primate agents randomly choose one of the neighboring grid-cells to move into. The decision of which of the 9 grid-cells (the location of the tree and its neighbors) is chosen at random (See UseStone submodel). When the global parameter *Treesdie* is set to true, locations of new trees added to the model are chosen at random (See age die grow submodel). Stochasticity is also observed in the calculation of the size of the fragment pounding tools that can detach from the Pounding Tools during use as it is drawn from a negative exponential distribution.

Collectives

The “assemblages” of Pounding Tools that accumulate in each grid cell can be thought of as a collective. At a broader scale, the entire set of Pounding tools could also be considered a collected as it is analogous to an “archaeological landscape”.

Observations

The number of Tree agents where it is possible to move tools to is recorded at every time step. The rest of the data are collected at the end of each simulation run.

**Details**

Initialization

Each simulation is initialized with N_a_ Primates, N_s_ sources, and N_t_ Trees. 30 unique simulations were run for each parameter combination. Sources are randomly assigned a quality value of 0, 25, 50, or 75. If the global parameter *Treesdie* is set to True, then each Tree is randomly assigned an age between 1 and 10000. The attribute *ts_born* is set to 0 for each tree. Please see behavior_space.py to run the same experiments reported in the Reeves et al. (Submitted). The simulations do not require any input files. The parameter values in the experimental design are shown in Table 1.

Table 1. Parameter values used to initialize the simulations reported in Reeves et al. 2021.

**Parameter Value(s)**

*Num_agents (N_a_ )..............................................*100

*Num_sources (N_s_).................................*10, 50, 500

*Num_Trees (N_t­_).....................*100, 500, 1000, 2000

*Treesdie….............................................*True, False

*search_rad............................................................*2

*max_ts…........................................................*75000

*iterations*.........................................................1-30

Data Collection

At the end of each run the simulation data is exported as an SQL database which contains five tables: run_data, environment, trees, sources, and tools. The run_data table contains the global parameters specific to that run. This includes, *run_id*, *datetime, n_times_steps, num_sources, num_trees, treesdie, search_rad,* and *tree_deaths*. The environment table records data on the number of Trees where it is possible to move and use a Pounding Tool at each time step. The attributed recorded in this table include *run_id, time_step, trees_available.* Remaining tables collect data on the different agent classes. The pos attribute of Trees, Pounding Tools and Sources is recorded as the attributes x and y. The trees table stores information on the Tree agents. This table collects data on the attributes, ­*run_id, id, x, y, ts_born, alive, ts_died, age.* Information on the sources is contained within the source table. Information on the following attributes is held in the source table: *run_id, id, x, y, rm_quality.*The tools table holds information on the following tool attributes: *run_id, id, parent_id, source_id, x, y, tool_size, original_size, active, rm_quality, ts_born, ts_died, n_uses.* Compile.py can be used merge all of the runs in to a single database.

**Submodels**

Primate Submodels

*Move*

To move, a Primate agent identifies its eight neighboring grid cells. Then, one of the eight identified grid cells is chosen at random. The primate attribute *pos* is then updated based on the (x,y) location of the chosen grid cell.

*CheckForTree*

The Primate agent identifies its location and its eight neighboring grid cells. Each grid cell is checked to determine if it contains a Tree. If a Tree is found, then the Primate attribute *NutTreeLoc* is updated with the location of the Tree. In the event that more than one Tree found within the neighborhood, then a Tree is chosen at random.

*CheckForStone*

The Primate agent identifies all the grid cells within a given radius set by the attribute *search_radius* of its current location. The Primate agent then determines if a Pounding Tool or source agent is contained found within one of these grid-cells (including its current location). The result of this check is then returned as a list.

*SelectStone*

If the length of the list returned from *CheckForStone* is greater than 0. Then the *SelectStone* method is triggered. The Primate agent will then select the object that is nearest to its location. If there are multiple objects that equally near, then then Primate will choose from these equally near objects at random.

*UseStone*

The Primate agent then moves to the location of the selected object (Source or Pounding Tool). If the chosen object is a Source agent, then a new Pounding Tool agent is generated at the location of the source. The size of the Pounding Tool is determined by drawing from a random normal distribution with mean and standard distribution equivalent to the size of the Pounding Tools documented in the Taï Forest ^3^. This newly generated tool is then moved to the location of the Tree on one of the Tree’s neighboring grid-cells. This choice is random. The Pounding Tool attribute *n_uses* is then updated from 0 to 1.

If a Pounding Tool is selected, then Pounding Tool is selected then the Primate agent will move the Pounding Tool to the Tree as described above. The attribute *n­_uses* is then updated by adding 1 to the current value in *n_uses.*

*BreakStone*

When BreakStone is triggered the size of the detached piece is determined by drawing from a random negative exponential distribution with a scale of 200. This is used to best approximate the size in grams of documented pounding tool fragments detached from Panda nut tools in the Tai Forest ^6^. The size of the resulting fragment can be no greater than half of the Pounding Tool attribute *Tool_size*. This allows the nature of the breakages to range from the frequent detachment of small pieces to raw examples where the tool splits in half. The size of the Pounding Tool being broken updated by subtracting the size of the fragment from its *Tool_size*. If ­­*Tool­­­­_size* dropped below 2000 then the Pounding Tool’s active attribute is updated from True to False.

A new Pounding Tool agent is then generated at the location of the Pounding Tool that has been broken with the determined size of the fragment. The size of the newly generated Pounding Tool is greater than 2000 then its attribute *active* is set to True, otherwise it is set to False.

Tree Submodels

*AmIavailable*

Each Tree keeps track of whether the distance between itself and a Source or a Pounding Tool is small enough to that a Primate would find a tool within its search radius should it encounter the Tree. This is determined by checking if a Pounding Tool or Source exists within a 3-grid cell radius of the Tree’s location. Pounding Tools that are no longer active are not included in this calculation (i.e *Tool_Size* is less than zero, *Active* is False).

*AgeDieGrow*

This method is only executed when the global parameter *treesdie* is True. At the end of each time-step the *age* of the Tree is updated by adding 1 to the current value for *age.* If *age* equals 10000 then the attribute *ts_died ­*is updated with a value corresponding to the current time step in the model run. The attribute *alive* is set to false. Then the data for the tree is added to the Trees table in the SQL database. The Tree is then removed from the simulation.

A location within a 10-grid cell radius of the previously removed Tree is then chosen at random. A new Tree is generated at this new location. The attribute *ts_born* is then set to the current timestep, *age* is set to 0 and *alive* is set to true.
