# Supplementary material for: Modeling a primate technological niche
Source: Sci Rep. 2021 Nov 30;11:23139. doi: 10.1038/s41598-021-01849-4 (PMC8632893; doi:10.1038/s41598-021-01849-4)
Supplement: Supplementary file 2 — Supplementary Information 2. [file 41598_2021_1849_MOESM2_ESM.pdf]

Modeling A Primate Technological Niche: Supplementary Tables and Figures

Table 1: Runs where no tool use events occurred. Note that the majority of runs that did not faciliate tool use are runs with only 100 Trees

Number of Sources	Number of Trees	Trees die	Number of runs
10	100	0	11
10	100	1	14

Table 2: A summary of number of Pounding Tool uses by raw material quality

Number of Sources	Number of Trees	Material fragility	Min N uses	Mean N uses	Max N uses	Max distance to source
100	10	0	1	22	412	11.704700
100	10	25	1	23	250	8.544004
100	10	50	1	26	171	10.816654
100	10	75	1	19	172	8.000000
100	100	0	1	22	531	15.652476
100	100	25	1	20	379	13.601470
100	100	50	1	22	247	15.811388
100	100	75	1	17	171	12.806249
100	500	0	1	18	543	15.297059
100	500	25	1	17	392	13.601470
100	500	50	1	16	251	13.892444
100	500	75	1	15	177	15.264337
500	10	0	1	31	585	19.209373
500	10	25	1	33	323	20.518285
500	10	50	1	24	272	17.204650
500	10	75	1	25	184	18.110770
500	100	0	1	30	653	30.083218
500	100	25	1	26	357	25.942244
500	100	50	1	25	299	23.769729
500	100	75	1	23	219	17.029386
500	500	0	1	24	766	27.018512
500	500	25	1	22	394	26.172505
500	500	50	1	20	326	24.186773
500	500	75	1	18	251	24.413111
1000	10	0	1	46	729	30.016662
1000	10	25	1	38	374	25.059928
1000	10	50	1	33	250	23.345235
1000	10	75	1	26	199	27.294688
1000	100	0	1	45	775	37.947332
1000	100	25	1	36	430	30.886890

Number of Sources	Number of Trees	Material fragility	Min N uses	Mean N uses	Max N uses	Max distance to source
1000	100	50	1	30	315	30.000000
1000	100	75	1	27	225	29.000000
1000	500	0	1	31	843	34.176015
1000	500	25	1	27	454	32.310989
1000	500	50	1	24	289	27.166155
1000	500	75	1	22	216	27.586228
2000	10	0	1	79	770	52.392748
2000	10	25	1	55	501	44.011362
2000	10	50	1	44	295	38.013156
2000	10	75	1	36	186	35.846897
2000	100	0	1	68	802	51.400389
2000	100	25	1	51	495	49.040799
2000	100	50	1	40	342	36.878178
2000	100	75	1	34	227	36.715120
2000	500	0	1	42	833	51.078371
2000	500	25	1	33	484	40.024992
2000	500	50	1	29	309	37.215588
2000	500	75	1	26	258	35.777088

Table 3: Runs where the repeated transport of tools did not result in an increase in the number of tool use opportunities

Number of sources	Number of trees	Trees die	Number of runs
10	100	0	28
10	100	1	18
10	500	0	11
10	500	1	2
10	1000	0	1
100	100	0	19
100	100	1	6
500	100	0	6
500	100	1	1

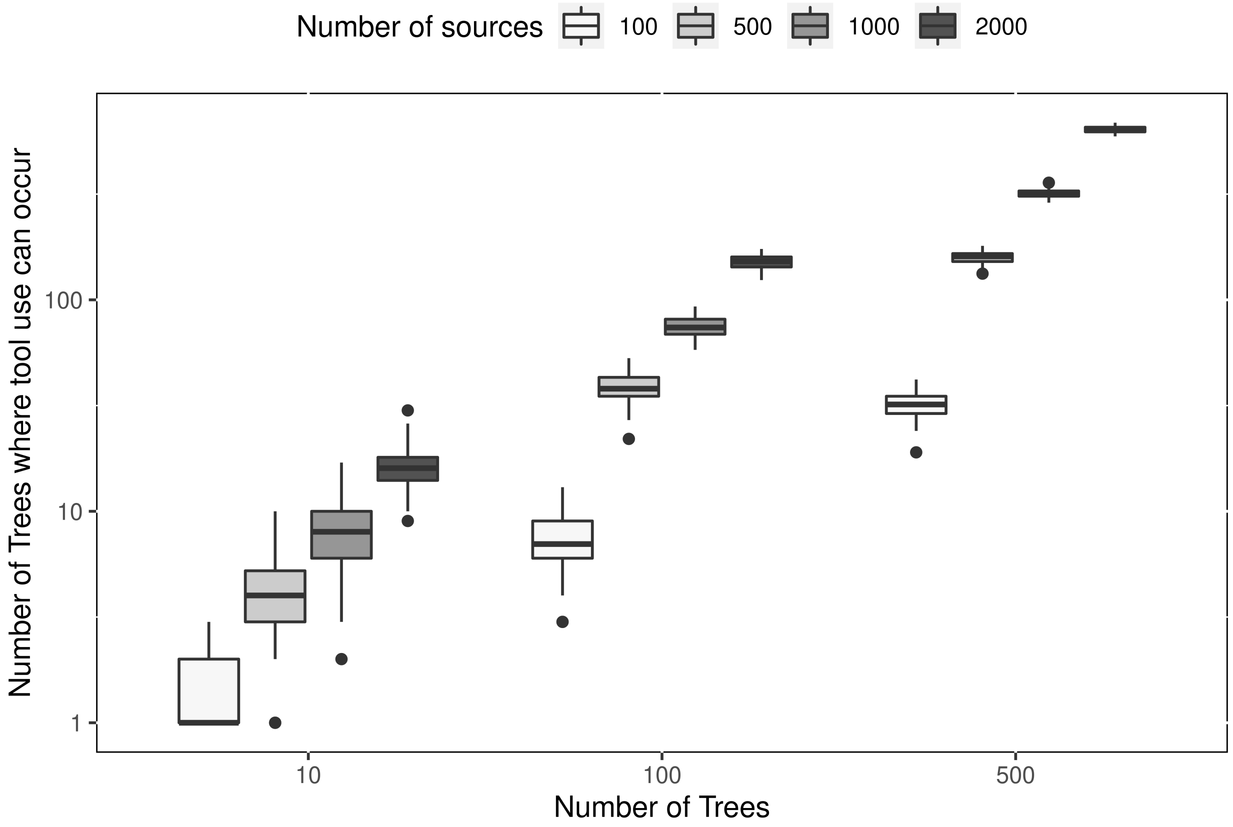

Figure 1: The relationship between the number of places where it is possible for a tool use event to occur and the number of trees and sources at the beginning of each model run. Increasing both the number of *Trees* and *Sources* included in the model has a positive effect on the number of places where tool-use can occur. Note that the Y axis is in log 10 scale

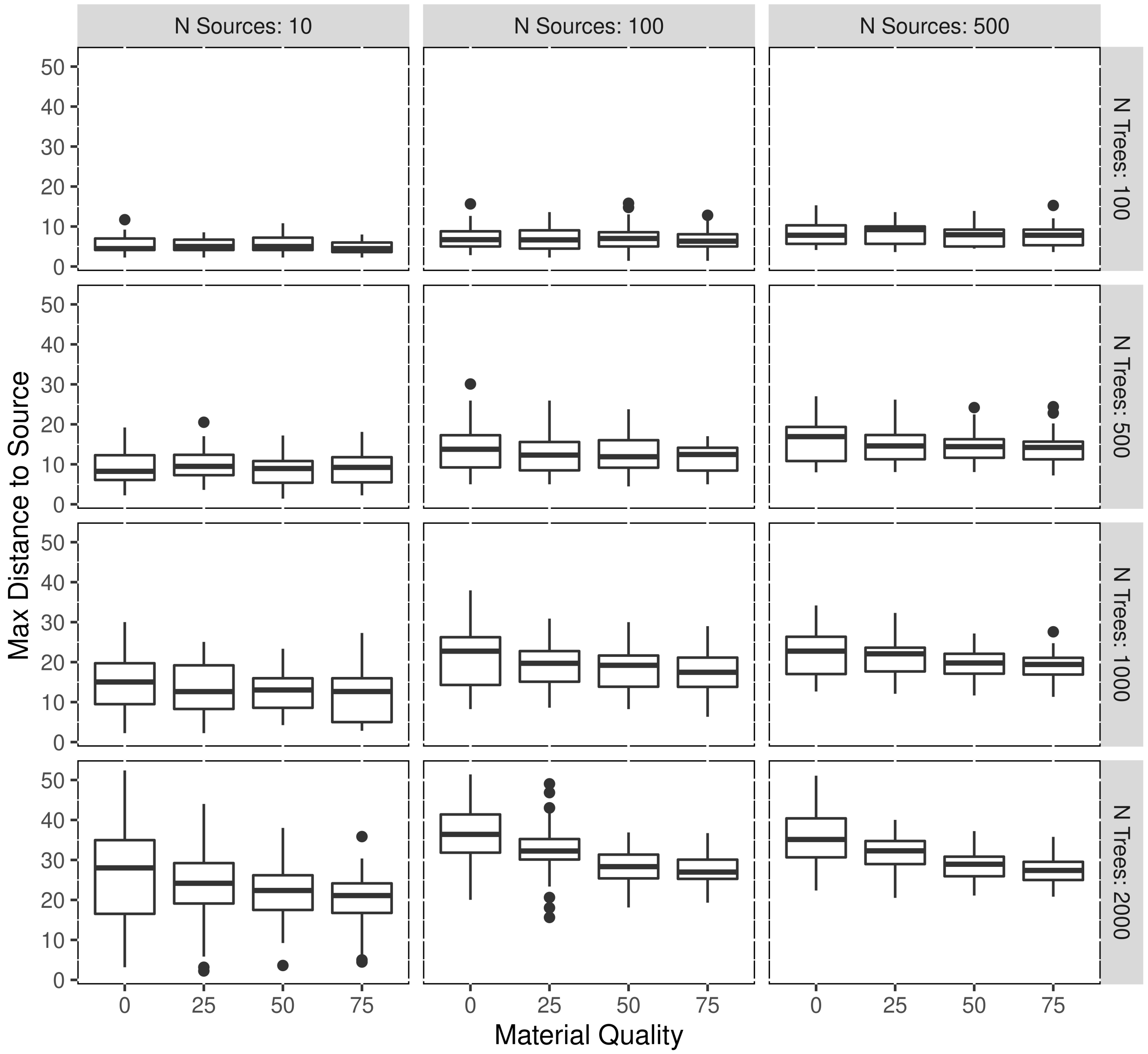

Figure 2: Both plots showing the maximum distance *Pounding Tools* were moved according to their fragility. When the number of Trees is low, material fragility has little influence on the maximum distance a *Pounding Tools* travel, this is due to the fact that there is little opportunity for tools to move substantial distances from their sources. However, as the number of *Trees* increases, so does the distance *Pounding Tools* can move from their *Source*. In cases where the number of *Trees* is great, the maximum distance tools can move is influenced by its raw material fragility. Note that a raw material quality of 0 reflects 25% chance of breaking whereas a raw material quality of 75 represents a 100% change of breaking.

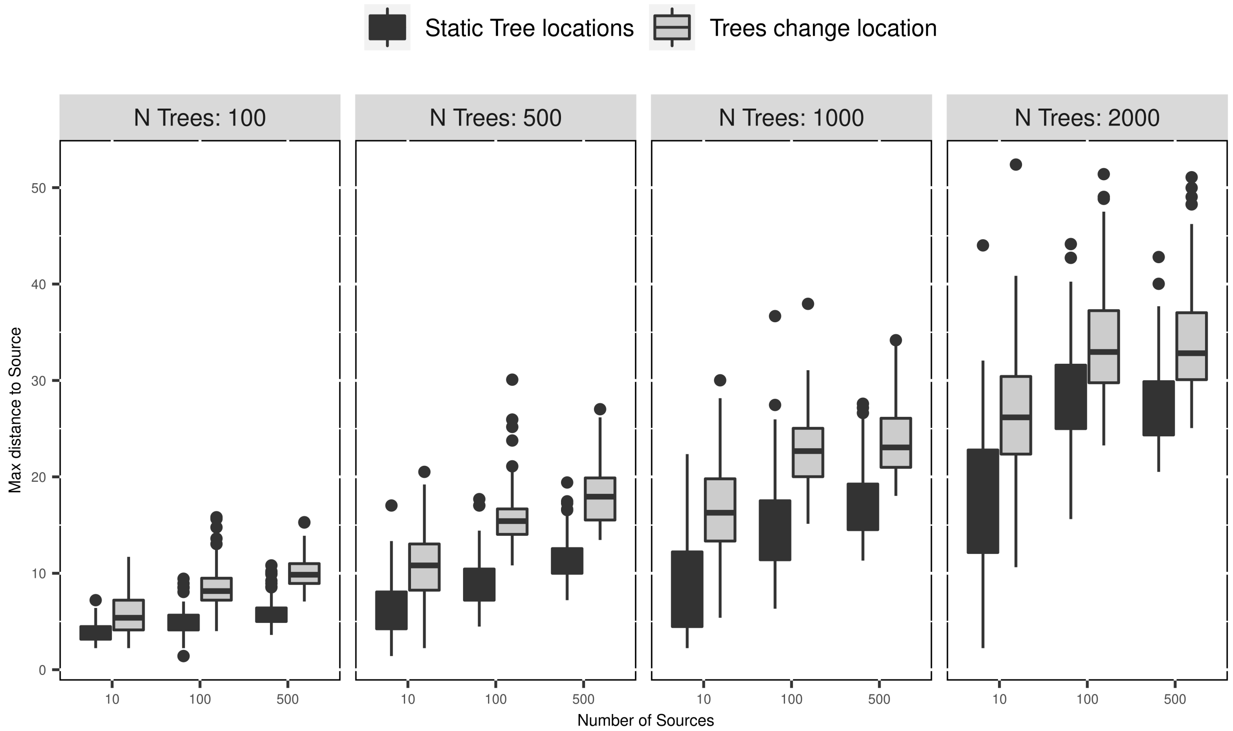

Figure 3: The effect *Tree* death and growth on the maximum distance tools can move from the source. When holding the number of *Trees* and *Sources* constant *Pounding Tools* the maximum distance a pounding tool can move is greater when *Trees* are able to change their location due to death and regrowth.

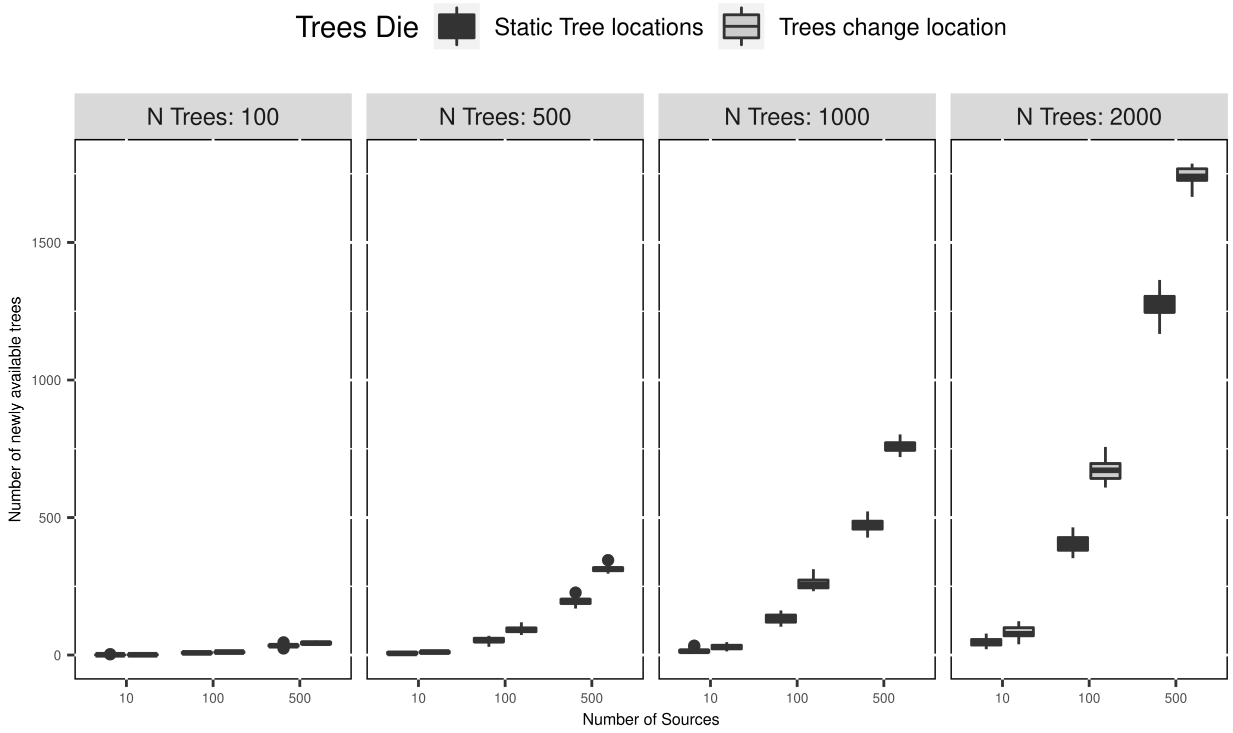

Figure 4: The effect changing tree locations on the number of trees that become accessible due to the transport of tools.

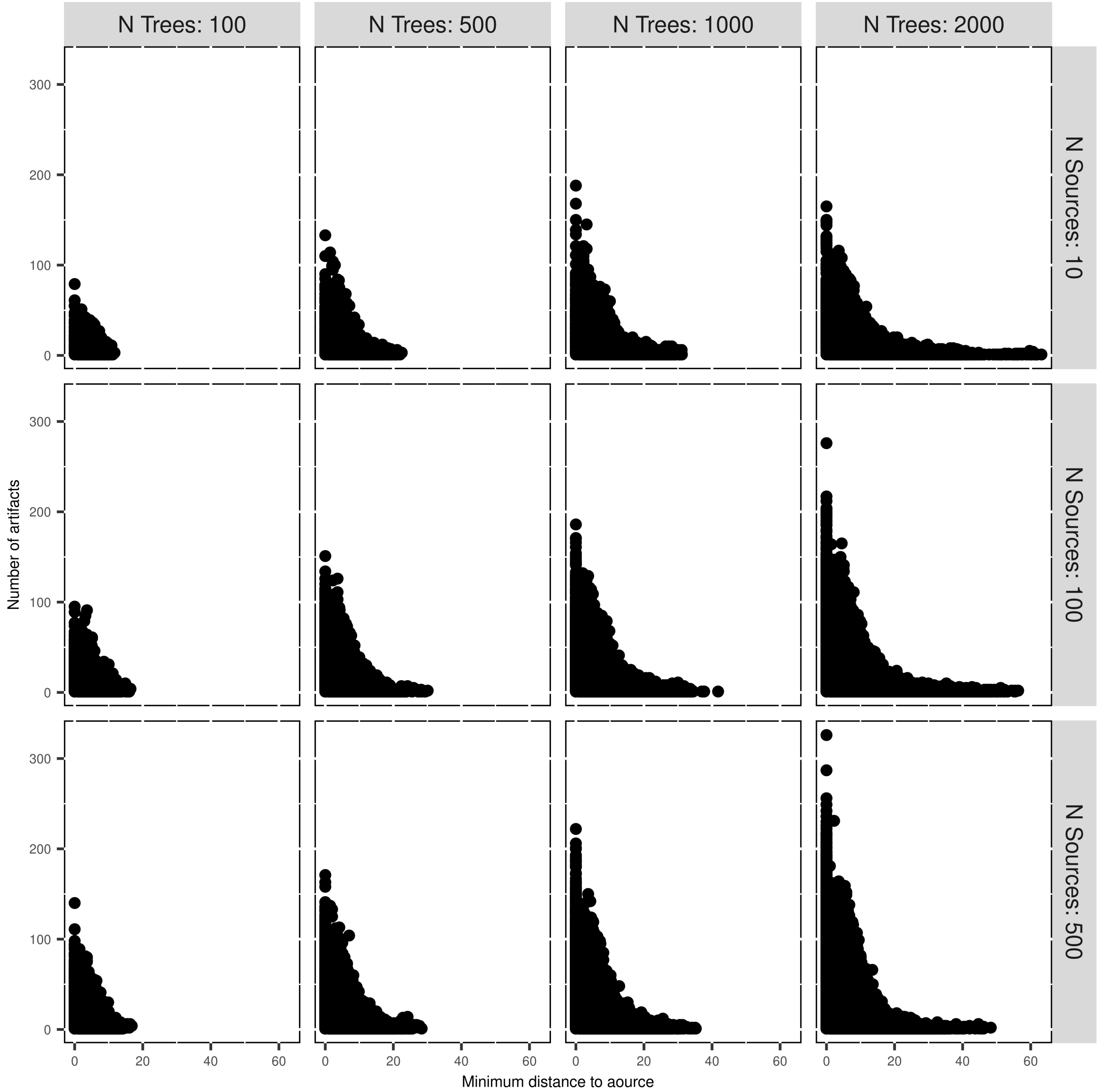

Figure 5: The relationship between the number of artifacts found in a grid cell and its distance to the nearest source. Note how the number of *Trees* attenuates the scale of the distance-decay relationship

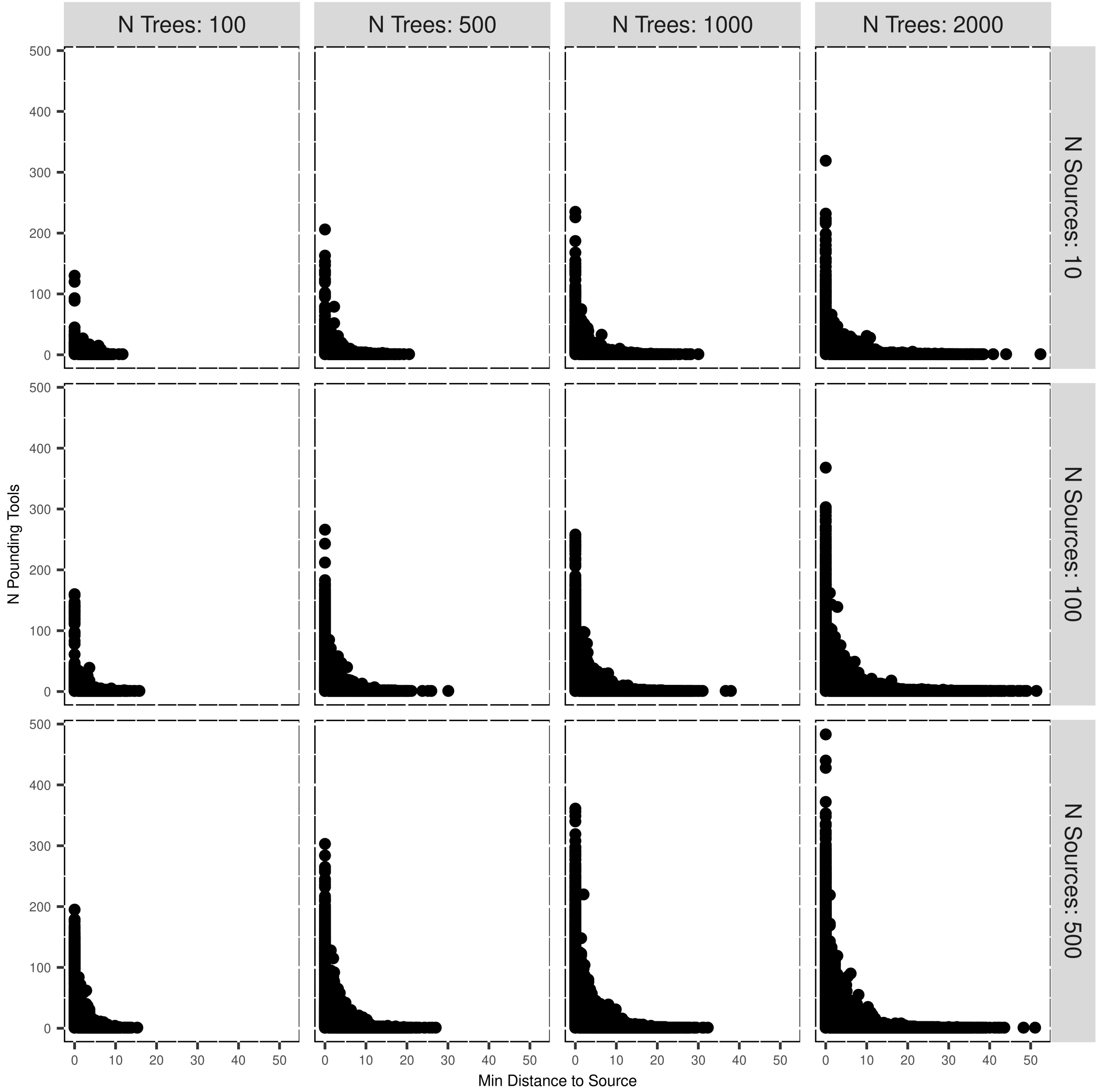

Figure 6: The relationship between the number of *Pounding Tools* found in a grid cell and its distance to the nearest source. Note how the number of *Trees* attenuates the scale and strength of this relationship

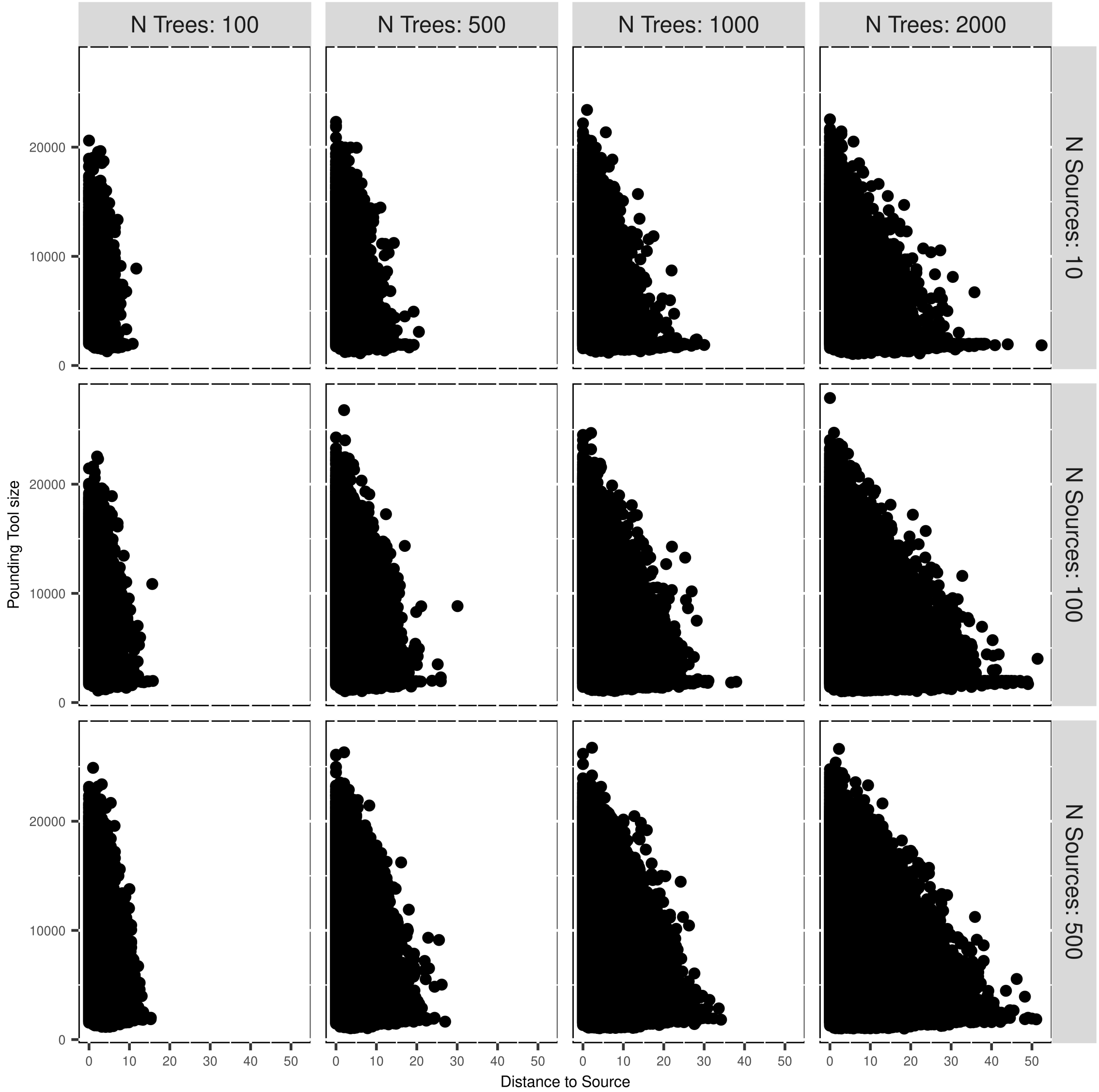

Figure 7: The relationship between the size of *Pounding Tools* and distance to their *Sources*. Note how the number of *Trees* attenuates the scale and strength of this relationship

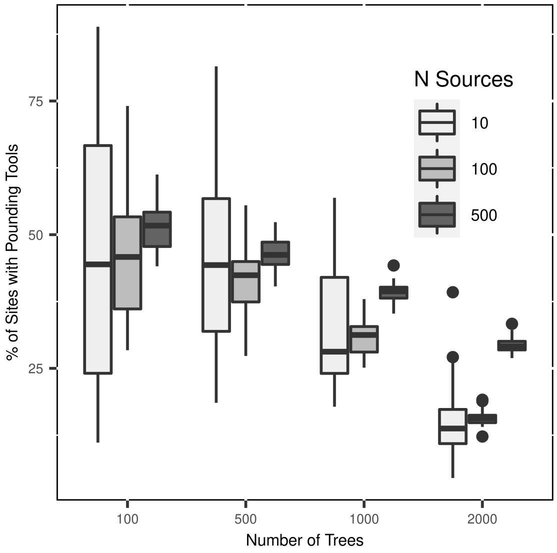

Figure 8: The effect of changing *Tree* locations on the representation of *Pounding tools* in the simulated material record in runs where Tree locations are static. Increasing the number of sources increases the percentage of assemblages that contain *Pounding Tools*. In comparison with figure 4 (right) in the main text, individual assemblages contain greater proportions of Pounding Tools
